# Supplementary material for: Transparency in peer review: Exploring the content and tone of reviewers’ confidential comments to editors
Source: PLoS One. 2021 Nov 29;16(11):e0260558. doi: 10.1371/journal.pone.0260558 (PMC8629260; doi:10.1371/journal.pone.0260558)
Supplement: Attachment — Submitted filename: Response to Reviewers - Transparency in peer review.pdf [file pone.0260558.s001.pdf]

Dear Dr. Baccini,

We thank you and the reviewers for your feedback and suggestions regarding our manuscript entitled, "Transparency in peer review: Exploring the content and tone of reviewers' confidential comments to editors" (Manuscript PONE-D-21-24629) by O'Brien, Artino, Costello, Driessen, and Maggio. We are pleased to resubmit our manuscript with revisions based on this feedback. In the table below, we provide a description of how we addressed each reviewer comment. These revisions are also noted within the document using track changes.

**Editor comments**	**Author response**	**Edit location in Marked Version**
Academic Editor		
From my part, I think that the data associated to your article do not permit a complete replication of your analysis. You should therefore add information to the data file available on Zenodo: 1) the classification of each article used for building Table 3 and Table 4 of the articles; 2) the recommendation of each referee and the final decision of the editor.	We have updated our data file on Zenodo to include the classification of each article type and the recommendation of each referee and the final decision of the editor. We also have created this as a .CSV file. We have provided the full data set, description of our codes, and sample quotes, so independent investigators have everything they need to replicate our findings. We prefer to allow others to make their own independent interpretations of the data and see how their classifications align with our results.	Zendo: https://doi.org/10.5281/zenodo.5128723
Journal Requirements		
Please ensure that your manuscript meets PLOS ONE's style requirements, including those for file naming. The PLOS ONE style templates can be found at<br><br>https://journals.plos.org/plosone/s/file?id=wjVg/PLOSOne_formatting_sample_main_body.pdf	We confirm that our manuscript meets style requirements	

https://journals.plos.org/plosone/s/file?id=ba62/PLOSOne_formatting_sample_title_authors_affiliations.pdf		
Please provide additional details regarding participant (author and reviewer) consent. In the ethics statement in the Methods and online submission information, please ensure that you have specified whether the need for consent to use reviews for research was specifically waived by the ethics committee.	The need for consent to use reviews was waived by the ethics committee. We added the statement "Our study was approved by the Ethical Review Board of the Netherlands Society for Medical Education, and the need for informed consent of reviewers and authors was waived. File number: NERN 2020.1.5. Date of decision 18-07-2020."	Lines 145-148
Thank you for stating the following in the Competing Interests section:<br><br>"I have read the journal's policy and the authors of this manuscript have the following competing interests: Erik Driessen (Editor-in-Chief), Lauren Maggio (Deputy Editor-in-Chief), and Anthony Artino (Associate Editor). Driessen and Maggio receive an honorarium for their editorial roles."<br><br>Please confirm that this does not alter your adherence to all PLOS ONE policies on sharing data and materials, by including the following statement: "This does not alter our adherence to PLOS ONE policies on sharing data and materials." (as detailed online in our guide for authors http://journals.plos.org/plosone/s/competing-interests). If there are restrictions on	We confirm that this does not alter our adherence to PLOS ONE policies and added the statement as requested.	Lines 502-505

sharing of data and/or materials, please state these. Please note that we cannot proceed with consideration of your article until this information has been declared.<br><br>Please include your updated Competing Interests statement in your cover letter; we will change the online submission form on your behalf.		
Please include your full ethics statement in the 'Methods' section of your manuscript file. In your statement, please include the full name of the IRB or ethics committee who approved or waived your study, as well as whether or not you obtained informed written or verbal consent. If consent was waived for your study, please include this information in your statement as well.	We added the statement "Our study was approved by the Ethical Review board of the Netherlands Society for Medical Education, and the need for informed consent of reviewers and authors was waived. File number: NERN 2020.1.5. Date of decision 18-07-2020."<br><br>Additionally, in the Data sources section of the Methods we provide the following statement, which comes from the author guidelines posted on PME's website: "PME believes in taking an evidence-based approach to publishing. PME notifies authors and reviewers in the author instructions, posted on the PME website, that the journal reserves the right to conduct research on all materials submitted to the journal to improve its processes."	Lines 145-148
Please review your reference list to ensure that it is complete and correct. If you have cited papers that have been retracted, please	We have reviewed our reference list to ensure it is complete and correct.	

include the rationale for doing so in the manuscript text, or remove these references and replace them with relevant current references. Any changes to the reference list should be mentioned in the rebuttal letter that accompanies your revised manuscript. If you need to cite a retracted article, indicate the article's retracted status in the References list and also include a citation and full reference for the retraction notice.		
Reviewer #1:		
The authors provide a first glimpse into the relationship between reviewer comments to editors and comments to authors. This type of investigation is long overdue and this work provides a first small step in this direction. Having anecdotally experienced myself that reviewer comments to the editor may diverge substantially from the comments to the authors, I have wondered how common this really is. While this is just an analysis of one journal, it is a start and it is reassuring that such divergence may not be all that common. I find the article well written and easy to follow. Methods are explained well and in sufficient detail.	Thank you.	
L186: "We coded the tone of comments as critical, constructive, supportive, or neutral"<br>There were no vicious or antagonistic, etc. comments	We appreciate this comment. Comments that could be interpreted as vicious or antagonistic would have been coded as critical. We did not find comments that we would	No changes

to the authors? Maybe, if this is a very collegial field, one needs to look at journals in more competitive fields to find differences in tone between comments to authors and comments to the editor? Should this surprising (to me) finding not be mentioned?	consider vicious, and we provided access to all data so that readers may review and judge for themselves. We agree that conducting this study in other fields and journals is important and a valuable next step. Since we did not begin this study expecting to find vicious comments, we prefer not to mention this as a surprising finding.	
L216: "Each article had 1 to 5 reviews." - further up it is mentioned that the journal policy requires two reviews?	Thank you for pointing this out. While two reviewers are required, the journal editors sometimes request additional reviews if they feel they need more information about the quality and/or importance of the manuscript. For articles with only one review, some articles were still under review and awaiting a second reviewer when we extracted the data. Others reflect situations in which an editor could only find one reviewer due, for example, to an article being in a niche area. In these situations, the editor may serve as a "second reviewer." We've added a sentence to the manuscript explaining why the number of reviews ranged from 1 to 5.	Lines 168-170 (Methods – Data source)<br><br>Lines 241-244 (Results)
Perhaps it is worth explicitly mentioning the overall/total incidence of diverging editor comments? Reader can calculate it, of course, from the tables, but assuming that no editor comments would mean alignment with author comments, I think it would be worth to explicitly mention the incidence of divergence in the text and maybe even in the	Thank you for this suggestion. There were 86 reviews in which the reviewer recommended reject. If we assume all with no comments are aligned, then there is 80% alignment in content (69/86) and 91% alignment in tone (79/86). We revised our abstract and main text to reflect the incidence in this way. We also added a	Lines 49-53 (Abstract)<br><br>Lines 219-222 (Methods – Data analysis)<br><br>Lines 364-366, 386 (Results)

abstract: after all, the main message of the work is that it may not be very common, so why not mention the incidence in the abstract?	sentence to the methods section explaining the decision to code reviews with no comments to the editor as aligned: “We coded all reviews that did not contain comments to the editor as aligned with comments to the author. This approach allowed us to calculate the overall incidence of alignment in reviews where reviewers recommended rejecting the manuscript.”<br><br>We also revised text in the results to clarify the assumptions and denominator: “We considered reviews with no comments to the editor as aligned in both content and tone. For content, we found that more comments aligned with the comments to the authors than did not (80% and 20%, respectively).”	
Reviewer #2:		
The paper is well-written. The research questions are clear described and answered and conclusions are appropriate. The authors see the limits of this study (analysis of comments of a single journal in a specific field) and suggests possible developments and further investigations.	Thank you.	
The authors fail to mention that the journal is open access. This is not a secondary fact with respect to the care of the transparency of the processes and provides an important key to	Thank you. We agree that the open-access nature of PME is an important facet of the journal, though we find it difficult to speculate about how this would affect our results. . PME does not have	No changes

the interpretation of the results. I suggest to add a paragraph on this and to add this among the "limits" of the study (the results would be the same in a closed access journal?)	an open peer-review process. Instead, the journal is free to authors to publish in and free to readers to read. We are unclear how or why this openness would impact on our results, especially because PME reviewers and editors receive similar instructions to those given to reviewers and editors at non open-access journals. As such, we have not added another paragraph.	
The dataset is available in Zenodo. Perhaps it would be better a csv and not an xls, according to the FAIR principles.	We have now updated our data set to CSV format according to FAIR principles	Zenodo: https://doi.org/10.5281/zenodo.5128723
